# In utero exposure to experimental maternal asthma alters fetal airway development in sheep

**DOI:** 10.1113/EP092502

**Published:** 2025-01-27

**Authors:** Sarah J. Hammond, Andrea J. Roff, Joshua L. Robinson, Jack R. T. Darby, Ashley S. Meakin, Vicki L. Clifton, Robert J. Bischof, Michael J. Stark, Megan J. Wallace, Andrew Tai, Janna L. Morrison, Kathryn L. Gatford

**Affiliations:** ^1^ Robinson Research Institute University of Adelaide Adelaide South Australia Australia; ^2^ School of Biomedicine University of Adelaide Adelaide South Australia Australia; ^3^ Early Origins of Adult Health Research Group, Health and Biomedical Innovation and Health Sciences University of South Australia Adelaide Australia; ^4^ Adelaide Medical School University of Adelaide Adelaide South Australia Australia; ^5^ Mater Medical Research Institute University of Queensland South Brisbane Queensland Australia; ^6^ Institute of Innovation, Science and Sustainability Federation University Australia Berwick Victoria Australia; ^7^ Department of Neonatal Medicine Women's & Children's Hospital North Adelaide South Australia Australia; ^8^ Respiratory and Sleep Medicine Women's & Children's Hospital North Adelaide South Australia Australia; ^9^ Department of Obstetrics and Gynaecology Monash University Clayton Victoria Australia; ^10^ The Ritchie Centre Hudson Institute of Medical Research Clayton Victoria Australia

**Keywords:** airway remodelling, asthma susceptibility, maternal asthma, pregnancy, respiratory development, sheep

## Abstract

The mechanisms linking maternal asthma (MA) exposure in utero and subsequent risk of asthma in childhood are not fully understood. Pathological airway remodelling, including reticular basement membrane thickening, has been reported in infants and children who go on to develop asthma later in childhood. This suggests altered airway development before birth as a mechanism underlying increased risk of asthma in children exposed in utero to MA. We hypothesised that in utero MA exposure would reduce airway diameter and increase airway‐associated smooth muscle area and reticular basement membrane thickness in neonatal offspring. Experimental MA was induced by maternal sensitisation followed by airway challenges with house dust mite before and during pregnancy. Lambs from control (*n* = 16) or MA (*n* = 26) ewes were delivered at ∼140 days gestation (term = 150 days), ventilated for 45 min, then humanely killed. Left lungs were inflation‐fixed, and cross‐sections of generation 2–5 airways were collected. Airway sections were stained with Haematoxylin and Eosin, Masson's Trichrome and Gordon and Sweet's histological stains for morphological analysis. Lamb body and lung weights were similar between groups (*P* > 0.5 and *P* > 0.7, respectively). Lambs that were exposed to MA had narrower airway diameters (*P* = 0.019) and thinner reticular basement membrane (*P* = 0.016) but similar airway‐associated smooth muscle area (*P* = 0.152) compared with unexposed control lambs. Our results demonstrate a potential mechanism for increased risk of asthma in children of mothers with asthma, independent of genetic risk or behavioural changes during pregnancy.

## Abstract (highlights)

- **What is the central question of this study?**
  Does in utero exposure to maternal allergic asthma impact airway development in fetal sheep?
- **What is the main finding and its importance?**
  This study provides evidence that in utero maternal asthma exposure alters fetal airway development, independent of genetics, as a potential mechanism for the underlying increased risk of asthma in children exposed to maternal asthma in utero.

## 1 INTRODUCTION

Asthma affects an estimated 260 million people globally and impacts 13%–25% of all pregnancies (Das et al., 2022). Maternal asthma (MA) is a risk factor for poor perinatal outcomes, such as pre‐eclampsia, preterm delivery and low birth weight (Murphy et al., 2013; Wang et al., 2014). Exposure to MA in utero is also a strong predictor of childhood asthma, with offspring of asthmatic mothers being three times more likely to develop asthma themselves than those born to non‐asthmatics (Lim et al., 2010). Importantly, many of the associated risks appear to be normalised when asthma symptom control is maintained during pregnancy (Grzeskowiak et al., 2016; Roff et al., 2025). Furthermore, the Managing Asthma in Pregnancy trial provided evidence that optimal asthma management during pregnancy significantly reduces the odds of doctor‐diagnosed asthma in preschool children (Morten et al., 2018). Such evidence suggests an independent contribution of MA exposure in utero for asthma pathogenesis, rather than being driven solely by genetic influence.

Pathological airway remodelling is a hallmark feature of asthma and includes thickening of airway‐associated smooth muscle, increased collagen deposition and thickening of the reticular basement membrane (RBM; underlying the airway epithelium), leading to reduced airway luminal diameter (Barrios et al., 2006). Although pathological airway remodelling was previously believed to be a response to airway wall injury (Halwani et al., 2010), more recent evidence suggests that airway remodelling can occur prior to development of asthma. Indeed, thickening of the airway epithelial RBM has been observed in 3‐year‐old children who went on to develop asthma by 8 years of age (Payne et al., 2003). Likewise, in the COPSAC_2000_ study (Bisgaard, 2004), airway obstruction and greater bronchial reactivity was evident at 1 month of age in children who developed asthma before the age of 13 years (Hallas et al., 2019).

We, therefore, hypothesised that in utero exposure to MA would impact airway development, reflective of the morphological changes associated with asthma‐driven pathological airway remodelling. Specifically, we predicted a decrease in airway diameter, increased airway‐associated smooth muscle and increased RBM thickness in progeny exposed to maternal asthma. This hypothesis was tested using an established preclinical model of maternal asthma during pregnancy in sheep (Clifton et al., 2016), whereby animals were sensitised to house dust mite (HDM; *Dermatophagoides pteronyssinus*), one of the most common respiratory allergens in humans (Bischof et al., 2008; Shah & Grammer, 2012).

## 2 MATERIALS AND METHODS

### 2.1 Animals and experimental design

This study was approved by the Animal Ethics Committee of the South Australian Health and Medical Research Institute (SAHMRI, approval SAM455.19). All investigators adhered to the ethical principles outlined by Grundy (2015) and the principles of the 3Rs (Tannenbaum & Bennett, 2015), and all experimental animal procedures were conducted in accordance with Australian guidelines (National Health & Medical Research Council, 2013). Animal studies were conducted at the SAHMRI Preclinical, Imaging and Research Laboratories, Gilles Plains, SA, Australia. The experimental design followed the ARRIVE guidelines (Kilkenny et al., 2010) throughout, to ensure unbiased, reproducible and transparently reported results.

This study forms one component of an overarching project investigating antenatal betamethasone as a rescue therapy for delayed fetal lung maturation in pregnancies complicated by asthma. Merino ewes (*n* = 65, 3 years of age, sourced from the SAHMRI Farm, Burra, SA, Australia) were paddock‐housed, with free access to water and seasonal pasture (supplemented with oaten hay as required). Detailed information on this model of experimental asthma, including protocols for allergen sensitisation and airway challenges, has been described previously (Bischof et al., 2003; Clifton et al., 2016). In brief, the present cohort of non‐pregnant ewes were randomly assigned to either control (*n* = 15) or MA (*n* = 50) groups (Robinson, Roff et al., 2024). Ewes allocated to the MA group were sensitised to HDM allergen (≥1.5‐fold increase in blood plasma HDM‐specific IgE, *n* = 26), followed by fortnightly airway challenges with HDM from 8 weeks before timed mating. Five non‐sensitised ewes were transferred to the control group and received fortnightly saline airway challenges. At ∼50 days post‐mating, pregnancies were confirmed by ultrasonography (control *n* = 11, MA *n* = 17), with fortnightly airway challenges continuing throughout gestation. The lung function of each ewe was assessed before mating and at days 62 and 132 after mating to confirm the development of the maternal asthma phenotype (Clifton et al., 2016).

Pregnant ewes in the MA group were randomised to receive intramuscular injections with saline or 11.4 mg of betamethasone (Celestone Chronodose 11.4 mg per dose, Schering Plough, Baulkham Hills, NSW, Australia) 48 and 24 h before delivery, consistent with the dosage regime used in clinical practice (Antenatal Corticosteroid Clinical Practice Guidelines Panel, 2015), to investigate effects on lung maturation. On day ∼140 of gestation (term, ∼150 days), maternal anaesthesia was induced with intravenous ketamine (7 mL kg^−1^) and diazepam (0.3 mL kg^−1^), then maintained in the ewe, hence the fetus, by inhalation of isoflurane (1.5%–2.5% in air; Lyppards, SA, Australia). Fetuses were exposed via caesarean section for catheterisation of the carotid artery and jugular vein, then intubated. The umbilical cord was tied and cut before delivery. Thereafter, ewes were humanely killed by intravenous infusion of sodium pentobarbitone (100 mg kg^−1;^ Virbac Australia, Peakhurst, NSW, Australia), and the lambs were towel‐dried and weighed. Anaesthesia of lambs was maintained with intravenous infusion of alfaxalone (5–15 mg kg^−1;^ Alfaxan‐CD RTU; Jurox Pty. Ltd, Rutherford, NSW, Australia) during a 45 min ventilation. Lambs were ventilated using a synchronised intermittent positive pressure ventilation + volume guarantee strategy using the Babylog 8000 Plus ventilator machine (Dräger Medical), with full details of the ventilation protocol described previously (Robinson, Roff et al., 2024). Lambs were then humanely killed with sodium pentobarbitone (20 mg kg^−1^) for collection of tissues.

### 2.2 Lamb airway analyses

Lamb lungs were collected and weighed, and the left lung instillation was fixed at 20 cmH_2_O with 4% paraformaldehyde. Following 24–30 h of fixation, airway cross‐sections were sampled (Wignarajah et al., 2002), starting directly after the first bifurcation into the left primary bronchus (generation 1). Consecutive samples were then collected post‐bifurcation from the largest branch systematically through to generation 5 (distal bronchi). Generation 1 samples were excluded from analysis owing to damage caused during lung fixation. One control lamb was also excluded owing to tissue loss at collection. Airways were embedded perpendicular to their length, serially sectioned (4 µm), and mounted onto de‐identified slides, ensuring blinding to the treatment group during subsequent analyses. Once histologically stained, slides were scanned and digitised using a ×40 objective lens (NanoZoomer 2.0‐HT, Hamamatsu Photonics K.K., Japan). Structural measures were conducted on all sections that were complete and oriented perpendicularly. A visible respiratory epithelium (pseudostratified columnar epithelium with cilia) and the presence of airway‐associated hyaline cartilage were used to confirm that cross‐sections were of airways rather than adjacent vasculature.

Airway luminal diameter was measured on Haematoxylin‐ and Eosin‐stained sections. For each airway, the major axis (the widest point of the airway lumen) was measured, followed by the minor axis (the widest point of the airway lumen intersecting the major axis at an angle of 90°; Figure 2a). The airway luminal diameter was calculated as the average of the lengths of the major and minor axes (Tzeng et al., 2007). The area of airway‐associated smooth muscle (ASM) and airway perimeter (measured at the basement membrane) was measured on Masson's Trichrome‐stained sections (Figure 3a), and ASM was expressed as a ratio to airway perimeter (Clifton et al., 2016). The thickness of RBM was measured on Gordon and Sweet's‐stained sections (Gordon & Sweets, 1936). Using a grid‐overlay method (Malmström et al., 2017), the thickness of RBM on each section was measured at points that intersected with a 1 cm × 1 cm grid overlaid on each digitised slide image (NDP.view2 2.8.24, Hamamatsu Photonics K.K., Japan) resulting in ∼20–200 measurements of RBM thickness per airway.

For all outcomes, the number of airway sections that were measured varied with airway size; generally two sections per sample for generations 2 and 3, and four per sample for generations 4 and 5. Measures were completed on all mounted sections and averaged to produce one data point per generation and lamb.

### 2.3 Statistical analyses

Initial analyses were performed to compare outcomes in lambs born to MA ewes treated with saline (*n* = 14 lambs) or antenatal betamethasone (*n* = 12 lambs). Airway morphology did not differ between these groups across generations (airway diameter, all generations, *P* = 1.000; airway‐associated smooth muscle area relative to airway perimeter, generations 2–4, *P* = 1.000, and generation 5, *P* = 0.718; reticular basement membrane thickness, generations 2–4, *P* = 1.000, and generation 5, *P* = 0.414). These two groups were therefore combined in subsequent analyses comparing control (*n* = 16) and MA‐exposed (*n* = 26) lambs. Data were tested for equal variance by the *F*‐test, and the Shapiro–Wilk test was used to determine normality. Effects of MA and airway generation on airway morphology were assessed by repeated‐measures mixed‐model ANOVA, with ewe included as a random factor to account for common maternal environment in twins. Where the interaction between maternal treatment and airway generation was below the threshold of *P* < 0.1, Bonferroni's *post hoc* test for multiple comparisons was used to assess the effect of MA within each airway generation. Statistical tests were performed in SPSS v.28 (IBM Corporation, Armonk, NY, USA), and data are presented as the mean ± SD, with *P* < 0.05 considered statistically significant.

## 3 RESULTS

### 3.1 Ewe and lamb phenotypes

At 140 days of gestation, control ewes weighed less than asthmatic ewes (*P* = 0.011; Table 1). Although exposure to MA did not affect absolute lamb birth weight (*P* = 0.523), birth weight as a percentage of ewe weight was greater in lambs born to control ewes (*P* = 0.044; Table 1). Exposure to MA also did not affect lamb lung weight in absolute terms (*P* = 0.724) or relative to lamb birth weight (*P* = 0.794; Table 1). Lamb absolute (*P* = 0.646) and relative (*P* = 0.869) liver weights, and brain‐to‐liver weight ratio (*P* = 0.321) were similar between groups (Table 1).

**TABLE 1:** Lamb and ewe characteristics.

Characteristic	Control	Asthmatic	*P*‐value
Number of ewes	11	17	–
Ewe body weight (kg)	73 (6)	80 (5)	**0.011**
Number of singleton lambs	5 (1 F, 4 M)	8 (5 F, 3 M)	–
Number of twin lambs	11 (6 F, 5 M)	18 (8 F, 10 M)	–
Lamb birth weight (kg)	4.67 (0.4)	4.49 (0.7)	0.523
Relative lamb birth weight (% of ewe weight)	6.14 (0.62)	5.61 (1.02)	**0.044**
Lamb lung weight (g)	157 (17)	153 (29)	0.724
Relative lamb lung weight (% of lamb birth weight)	33.8 (2.5)	34.4 (4.1)	0.794
Lamb liver weight (g)	117 (19)	114 (21)	0.646
Relative lamb liver weight (% of lamb birth weight)	24.7 (2.6)	25.5 (3.8)	0.869
Brain to liver weight ratio (g:g)	0.49 (0.07)	0.53 (0.15)	0.321

*Note*: Results were analysed by mixed‐model ANOVA. Data are the mean (SD) unless otherwise indicated. Statistical significance (*P* < 0.05) is shown in bold.

Abbreviations: F, female; M, male.

### 3.2 Airway luminal diameter

**FIGURE 1:**
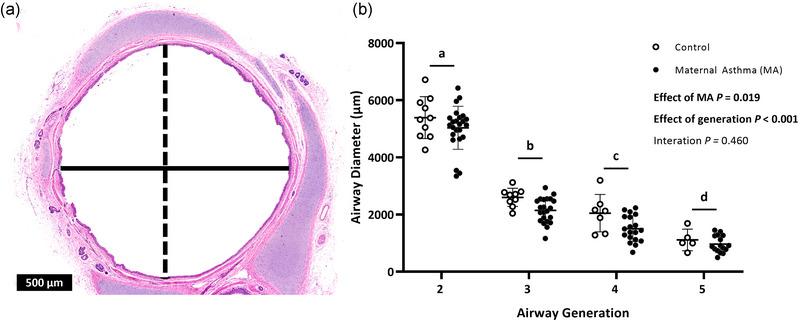
(a) Representative image of a generation 3 airway (×3.5 magnification) stained with Haematoxylin and Eosin, with major (dashed line) and minor (continuous line) axes indicated. The black scale bar represents 500 µm. (b) Generation 2–5 airway diameters in lambs born to control (open symbols) or asthmatic (MA) ewes (filled symbols). Bonferroni's *post hoc* test for multiple comparisons was used to determine which airway generations differed. Generations lacking a common lowercase letter (a, b, c, d) differ (*P* < 0.05). Symbols show data from individual animals, and bars indicate the mean (SD). Data were analysed by repeated‐measures mixed‐model ANOVA. Abbreviation: MA, maternal asthma.

Overall, MA‐exposed lambs had narrower airway diameters (*P* = 0.019; Figure 1), which decreased between each successive airway generation (*P* < 0.001). However, airway diameter relative to lamb body weight at post‐mortem examination was similar between groups (*P* = 0.179).

### 3.3 Airway‐associated smooth muscle

**FIGURE 2:**
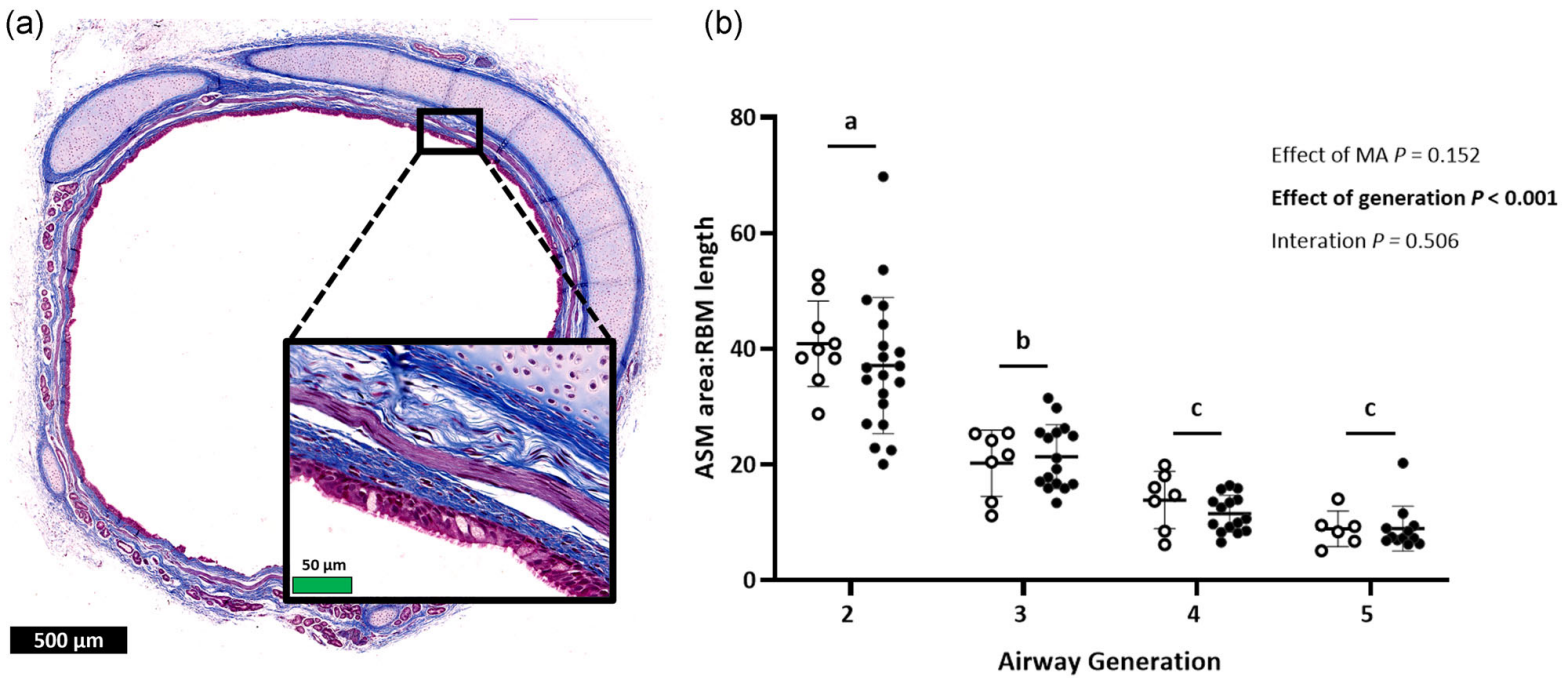
(a) Representative image of a generation 3 airway stained with Masson's Trichrome, indicating ASM bundles (outlined in yellow) and airway perimeter (RBM; traced in cyan) measures. Black and green scale bars represent 500 and 50 µm, respectively. (b) Generation 2–5 ratio of smooth muscle area to basement membrane length in lambs born to control (open symbols) or asthmatic (MA) ewes (filled symbols). Bonferroni's *post hoc* test for multiple comparisons was used to determine which airway generations differed. Generations lacking a common lowercase letter (a, b, c) differ (*P* < 0.05). Symbols show data from individual animals, and bars indicate the mean (SD). Data were analysed by repeated‐measures mixed‐model ANOVA. Abbreviations: ASM, associated smooth muscle; MA, maternal asthma; RBM, reticular basement membrane.

Airway‐associated smooth muscle area relative to airway perimeter was not affected by MA exposure (*P* = 0.152; Figure 2) and differed between airway generations (*P* < 0.001), decreasing in each generation from generation 2 to generation 4 (generation 2 vs. 3, *P* < 0.001; generation 3 vs. 4, *P* = 0.022) but not differing between generations 4 and 5 (*P* = 0.138).

### 3.4 Airway reticular basement membrane

**FIGURE 3:**
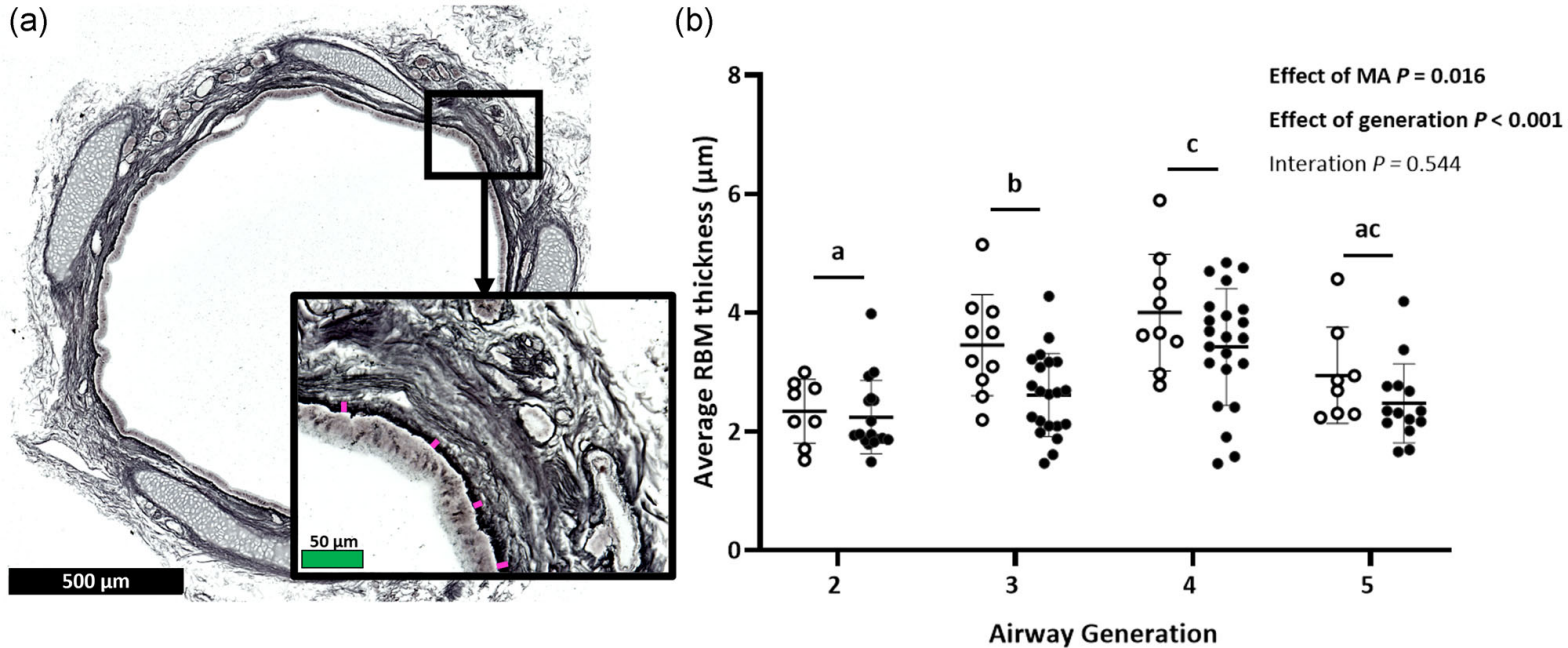
(a) Representative image of a generation 5 airway (×5 magnification) stained with Gordon and Sweet's reticulin stain, with magnified inset (×40 magnification) for visualization of the RBM (darkly stained, with pink lines indicating RBM location and thickness). Black and green scale bars represent 500 and 50 µm, respectively. (b) RBM thickness of airway generations 2–5 in lambs born to control (open symbols) or asthmatic (MA) ewes (filled symbols). Bonferroni's *post hoc* test for multiple comparisons was used to determine which airway generations differed. Generations lacking a common lowercase letter (a, b, c, d) differ (*P* < 0.05). Symbols show data from individual animals, and bars indicate the mean (SD). Data were analysed by repeated‐measures mixed‐model ANOVA. Abbreviations: MA, maternal asthma; RBM, reticular basement membrane.

Airway RBM was thinner in MA‐exposed lambs than in control lambs (*P* = 0.016) and differed with airway generation (*P *< 0.001; Figure 3). Lamb airway RBM was similar (*P* = 0.208) and thinnest in generations 2 and 5. Generation 2 was thinner than generations 3 and 4 (*P* = 0.017 and *P* < 0.001, respectively), and generation 3 was thinner than generation 4 (*P* = 0.013), which had the thickest RBM.

## 4 DISCUSSION

Our study provides the first evidence that in utero MA exposure alters fetal airway development, independent of genetics. Progeny airway diameter near term was narrower and RBM was thinner in lambs whose mothers had mild experimentally‐induced asthma in comparison to near‐term lambs delivered from mothers without asthma. Interestingly, although no evidence of growth restriction was observed in this cohort, with no difference in absolute body or lung weight between groups, airway diameter when normalised to body weight was similar. This finding suggests that the impact of maternal asthma on body weight overall contributes to effects on airway size. Nonetheless, the evidence generated in the present study highlights a significant change in airway structure that might underlie the increased risk of developing asthma in children whose mothers have asthma.

The reduced airway diameter in lambs whose mothers had asthma is consistent with the lower airway luminal diameter observed in human asthmatics (Bates, 2016), and is likely to be functionally important, because decreased airway diameter increases airway resistance (Bates, 2016). By increasing airway resistance, narrower airway diameter at birth might contribute to the poorer lung function evident in some infants who go on to develop asthma (Hallas et al., 2019). Children whose mothers have asthma have a 76% greater risk of asthma themselves (Roff et al., 2024), and our results suggest that airway remodelling induced by in utero MA exposure might contribute to this elevated risk of childhood asthma. Directly testing this hypothesis will require an assessment of postnatal lung function and airway morphology in the progeny of ewes with experimental asthma and controls. In contrast to the effects of MA on airway diameter, the thinner RBM in lambs from asthmatic ewes is inconsistent with the pattern of pathological airway remodelling in humans with asthma, which is characterised by RBM thickening (Palmans et al., 2002). It is unclear how RBM thickening in human asthmatics contributes to disease progression, particularly given that RBM thickness is not correlated with disease severity (Benayoun et al., 2003; Jeffery, 2001). Interestingly, RBM thickening has been proposed to be an adaptive response that might serve to protect lung function in asthma sufferers, providing resistance against airway narrowing and air trapping (Evans et al., 2010; Milanese et al., 2001). If this is the case and should the thinner RBM observed in lambs exposed to in utero MA persist, airway function might be impaired in later life, particularly in combination with narrower airway diameters. In addition to the possibility of contributing to asthma onset, narrower airways in individuals exposed to MA could also be implicated in the increased risks of other respiratory diseases (e.g., respiratory distress syndrome, respiratory tract infections and transient tachypnoea of the newborn) in the progeny of asthmatic mothers (Robinson, Gatford et al., 2024). Investigating the effects of in utero exposure to MA on more distal bronchi and bronchioles should also be considered. In humans, the bronchioles are thought to undergo extensive remodelling early in asthma progression, in a similar pattern to that seen in larger airways (Hastie et al., 2010).

Strengths of the present study include our use of a validated preclinical model of asthma (Bischof et al., 2003, 2008), which we have assessed previously during pregnancy (Clifton et al., 2016). Our use of sheep in this investigation, rather than other preclinical models, offers several advantages (Meeusen et al., 2009). Sheep represent a cost‐effective and accessible model of similar size and lung structure to humans. The bronchial tree is characterised by symmetrical, dichotomous branching in both sheep and humans (Morrison et al., 2018), which is in contrast to the asymmetrical, monopodial branching patterns of rodent lungs (Meeusen et al., 2009). Sheep, like humans, exhibit a clear transition in lung airway architecture from bronchi to bronchiole, a feature that is not identifiable in some rodent species, with the mouse often lacking respiratory bronchioles entirely (Suarez et al., 2012). Importantly, for studies investigating the impact of in utero exposure and interventions, the timing of lung development relative to birth is also comparable in sheep and humans. The embryonic stage, which represents the beginning of lung development, starts by week 3 of gestation in sheep and week 4 in humans (Schittny, 2017). Progression through the subsequent pseudo‐glandular, canalicular and saccular stages of lung development is sequentially similar in humans, sheep and rodents (Lock et al., 2013; Schittny, 2017). Humans and sheep continue to develop past the saccular stage during gestation, and term birth occurs during the alveolar stage. In contrast, rodent lungs are still at the saccular stage of development at term, progressing through to the alveolar stage postnatally (Lewin & Hurtt, 2017; Lock et al., 2013; Schittny, 2017). These similarities in developmental trajectory and lung structure between humans and sheep mean that our findings are highly translatable to the clinical context.

We also acknowledge the limitations of this study. Notably, the maternal asthma phenotype that has been described in detail elsewhere (Robinson, Roff et al., 2024) was milder than in our previous work in this model (Clifton et al., 2016) and did not impact markers of neonatal lung maturation or function. Given the mild maternal phenotype in this experimental cohort and the evidence that milder and better‐controlled maternal asthma is associated with better perinatal outcomes and lower risk of asthma in progeny (Roff et al., 2024), it was surprising that MA impacted fetal airway development in the present cohort. Although further studies are needed to elucidate the mechanisms underlying such changes, allergen sensitisation in neonatal lambs exposed to maternal asthma is not a viable explanation, because antibodies do not cross from maternal to fetal circulations via the intact sheep placenta (Poitras et al., 1986). Consistent with this, in our previously published study using the same model, HDM‐specific IgE antibodies were absent from lamb plasma samples (Wooldridge et al., 2019). This lack of HDM‐specific IgE in fetuses from ewes allergic to HDM is also consistent with HDM proteins not crossing the ovine placenta, because the fetal sheep immune system is developed enough to mount an allergen‐specific response by mid‐gestation (Fahey & Morris, 1978). Thus, we speculate that maternal inflammation rather than sensitisation to HDM in the fetal period is likely to be driving the changes observed. More studies are needed to confirm this hypothesis and characterise any long‐term outcomes of this mild MA on postnatal lamb airway structure and lung function. The impacts of moderate and severe asthma on airway development in our sheep model of MA also require further investigation.

## 5 CONCLUSION

Overall, our findings provide new evidence that MA during pregnancy impacts fetal airway development, even in the context of a mild MA phenotype. Progeny of ewes with experimental asthma had narrower airways and thinner RBM than near‐term neonates. If this also occurs in human pregnancies, altered airway development might contribute to the increased risk of asthma observed in children of asthmatic mothers (Lim et al., 2010; Roff et al., 2024). Indeed, our observation that even mild maternal asthma can adversely impact fetal lung development emphasises the need to optimise the management of asthmatic mothers during pregnancy to protect the long‐term respiratory health of offspring.

## AUTHOR CONTRIBUTIONS

Kathryn L. Gatford, Michael J. Stark, Megan J. Wallace, Janna L. Morrison, Robert J. Bischof, Vicki L. Clifton and Andrew Tai were responsible for the conception and design of the experiments. Sarah J. Hammond, Andrea J. Roff, Joshua L. Robinson, Jack R. T. Darby, Ashley S. Meakin, Kathryn L. Gatford, Michael J. Stark and Janna L. Morrison performed the experiments. Sarah J. Hammond, Kathryn L. Gatford, Janna L. Morrison, Megan J. Wallace and Andrew Tai acquired, analysed and interpreted data. Sarah J. Hammond, Kathryn L. Gatford and Janna L. Morrison drafted this article. All authors approved the final version of the manuscript and agree to be accountable for all aspects of the work in ensuring that questions related to the accuracy or integrity of any part of the work are appropriately investigated and resolved. All persons designated as authors qualify for authorship, and all those who qualify for authorship are listed.

## CONFLICT OF INTEREST

None declared.

## Data Availability

The data that support the findings of this study are available from the corresponding author upon reasonable request.
